# Squamous cell carcinoma of unknown primary site presenting with an abdominal wall lesion as the primary symptom: A case report and review of the literature

**DOI:** 10.3892/ol.2015.3520

**Published:** 2015-07-21

**Authors:** YINGLI ZHANG, BO CHEN, JIANQING ZHU, LU CHEN

**Affiliations:** Department of Gynecological Oncology, Zhejiang Cancer Hospital, Hangzhou, Zhejiang 310022, P.R. China

**Keywords:** squamous cell carcinoma, carcinoma of unknown primary site, diagnosis, chemotherapy, supportive care, prognosis

## Abstract

Squamous cell carcinoma of unknown primary site (SC CUP) is a rare malignant tumor, and its histogenesis and appropriate treatment are unclear. To the best of our knowledge, this type of carcinoma with abdominal wall lesions as the primary presenting symptom 3 months after laparoscopic surgery, has not been previously described in the literature. In the present study, a postmenopausal 54-year-old female patient was diagnosed with pain from the right abdominal puncture site 3 months after laparoscopic unilateral left salpingo-oophorectomy at a local hospital, at which time the left ovary and Fallopian tube were free of malignant tumor. Computed tomography (CT) imaging showed a subcutaneous nodule with a size of 6.2×3.3 cm. A wide excision of the lesion with safety margins and repair of the abdominal wall was performed, and the histopathological results and various investigations lead to the diagnosis of metastatic well-differentiated SC CUP. The patient underwent three surgeries and eight cycles of Taxol and cisplatin/carboplatin chemotherapy, and received a total of 10.8 Gy palliative radiation. However, the patient succumbed to intestinal bleeding, thrombocytopenia and multiple organ failure with pelvic recurrence and liver metastases at 10 months post-diagnosis. The prognosis of SC CUP, particularly with multiple metastases, is extremely poor. Although chemotherapy, surgery and radiotherapy have a certain role in the treatment, no regimen has been established as a standard therapy and palliative care could be recommended.

## Introduction

Carcinoma of unknown primary site (CUP) is defined as metastatic cancer that is present in the absence of an identifiable primary tumor site, even following thorough clinical examinations and diagnostic studies. CUP is a heterogeneous group of nosological entities based on histological, clinical, therapeutic and prognostic characteristics, and accounts for 3–5% of all malignancies. According to its histopathological characteristics, CUP can be classified into four major subtypes: well- or moderately-differentiated adenocarcinomas; undifferentiated or poorly-differentiated adenocarcinomas or carcinomas; squamous cell carcinomas (SCC); and undifferentiated neoplasms. SCC accounts for 15% of CUP, conferring a poor prognosis, with a median survival time of only 6–9 months (1,2), and these patients are usually treated with aggressive multimodal therapy similar to patients with locally advanced head and neck cancer. The present study describes a case of SC CUP with abdominal wall lesions as the primary symptom, 3 months after laparoscopic unilateral left salpingo-oophorectomy.

## Case report

In March 2013, a 54-year-old female (gravida 2, para 1) presented with a left ovarian cyst measuring 7 cm during a regular medical examination in a local hospital. There was no history of loss of appetite, weight loss or any other bladder- or bowel-associated symptoms. A clinical evaluation was made with regard to ultrasound and serological markers. Ultrasound examination showed a left ovarian cyst of 7×6×5 cm, with a normal uterus, right ovary and fallopian tubes. The serum level of carbohydrate antigen 19-9 (CA19-9) was above normal at 94.4 U/ml (reference range, 0–35.0 U/ml). Cancer antigen 125 (CA125), SCC antigen (SCC-Ag), α-fetoprotein (AFP), carcinoembryonic antigen (CEA) and neuron-specific enolase (NSE) levels were all within the normal ranges. A provisional diagnosis of an ovarian endometrioid cyst was made.

The patient underwent a laparoscopic unilateral left salpingo-oophorectomy. The left ovarian mass, adhering to the broad ligament, was removed along with the fallopian tube. The cyst broke upon separating the adhesion and ‘chocolate-like’ fluid flowed into the pelvic cavity. The cyst wall was removed through the laparoscopic puncture hole on the right abdominal wall. There were no other abnormalities in the abdominal cavity, which was irrigated with normal saline several times prior to closure. Histopathological analysis identified the mass as an endometrioid cyst.

**Figure 1.**
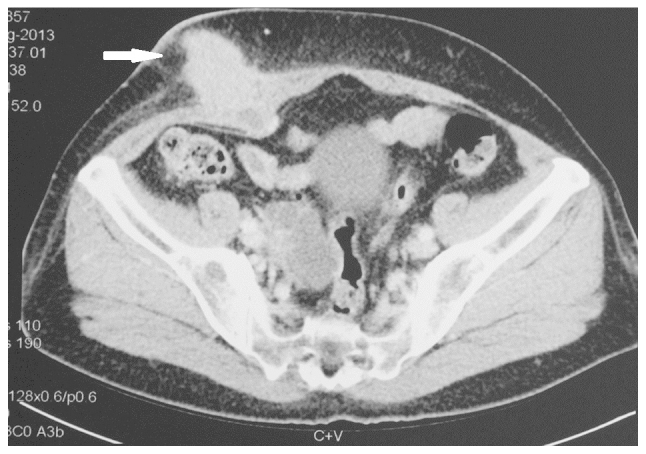
Computed tomography scan of a subcutaneous nodule (6.2×3.3 cm).

**Figure 2.**
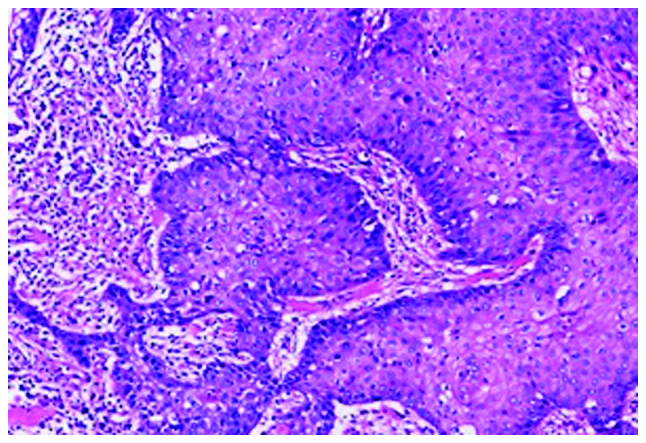
Photomicrograph showing the metastatic, well-differentiated squamous cell carcinoma of the right abdominal wall. The tumor cells are irregular, with large and hyperchromatic nuclei (hematoxylin and eosin stain; magnification, ×100).

The patient reported pain from the right laparoscopy puncture site at 3 months post-surgery. A 6.2×3.3-cm subcutaneous nodule was found by computed tomography (CT) imaging (Fig. 1). Furthermore, the serum CA19-9 level was level further increased at 113.5 U/ml. The CA125, SCC-Ag, AFP, CEA and NSE levels remained within the normal reference ranges. A wide surgical excision of the lesion with safety margins was performed and the abdominal wall was repaired with a reinforcing polypropylene mesh on September 2, 2013. Histopathologically, the surgical specimen contained a metastatic well-differentiated SCC with the following immunohistochemical staining results: p63(++), cytokeratin (CK)5/6(++), CK7(partially+), CK20(−), high molecular weight CK(++) and E-cad(+), with a Ki-67 of 10% (Fig. 2).

**Figure 3.**
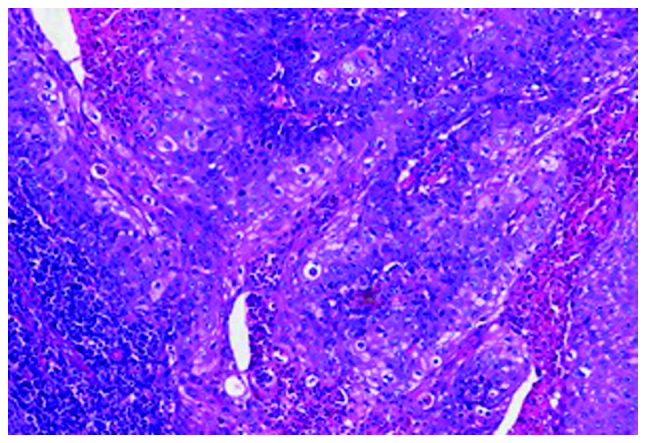
Photomicrograph showing the poorly-differentiated squamous cell carcinoma of the right inguinal lymph node. The tumor cells are polygonal, with necrosis in the center of the tumor cell nests (hematoxylin and eosin stain; magnification, ×100).

Due to the malignant nature of the disease, the patient was admitted at the Zhejiang Cancer Hospital (Hangzhou, China) for immediate further treatment on October 15, 2013. The first aim was to identify the primary site using a human papilloma virus test, cervical biopsy, gastroscopy examination and laryngoscopic examination, all of which were negative. Histological materials that were prepared at the time of the first surgery were examined section by section, and were all found to be without malignancy. Positron emission tomography (PET)/CT imaging was performed and revealed hypermetabolic lesions in the right pelvic cavity (tumor size, 4.3×2.6 cm) and right inguinal lymph node (tumor size, 0.5×0.9 cm). A biopsy of this inguinal lymph node revealed moderately-differentiated metastatic SCC (Fig. 3). The final treatment decision following a multidisciplinary discussion was to perform a laparotomy with a hysterectomy, right salpingo-oophorectomy, omentectomy, pelvic lymph node excision and multiple biopsies of the peritoneum. The surgical exploration found no ascites, a normal right ovary, a normal appendix, and a twisted and thickened right Fallopian tube adhering to the right ureter and rectal wall. A hard nodule ~3 cm in diameter was present on the left laparoscopy puncture site and left uterine broad ligament area, respectively. The nodule on the left uterine broad ligament area formed a compact adhesion to the rectum and left ureter.

**Figure 4.**
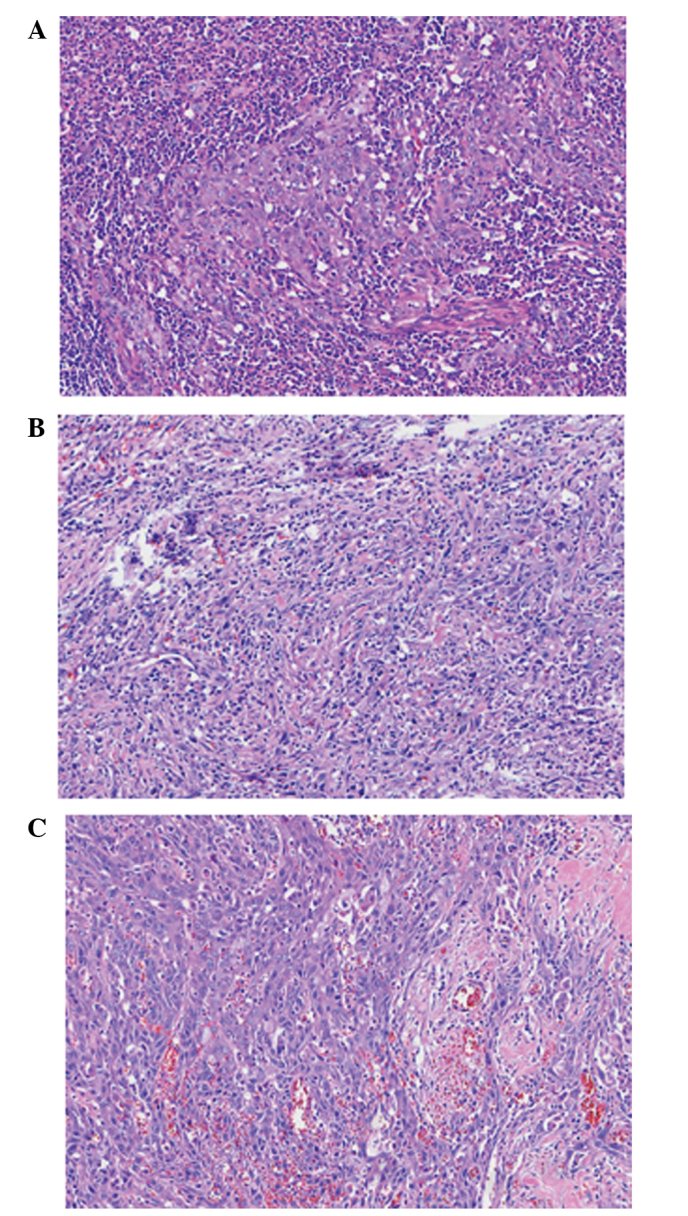
Photomicrograph showing the poorly-differentiated squamous cell carcinoma of the (A) left broad ligament, (B) the pouch of Douglas and (C) the left abdominal wall. The tumor cells varied in size, and showed irregular nuclei and distinct nucleoli (hematoxylin and eosin stain; magnification, ×100).

Immunohistochemical analysis showed poorly-differentiated invasive or metastatic SCC in the left broad ligament, the pouch of Douglas and the left lower abdominal wall (Fig. 4), which stained positive for the SC markers CK5/6 and p63, slightly positive for the glandular epithelial cell marker CK7, and was negative for CK20. The right ovary, right Fallopian tube and pelvic lymph nodes were free of tumor.

**Figure 5.**
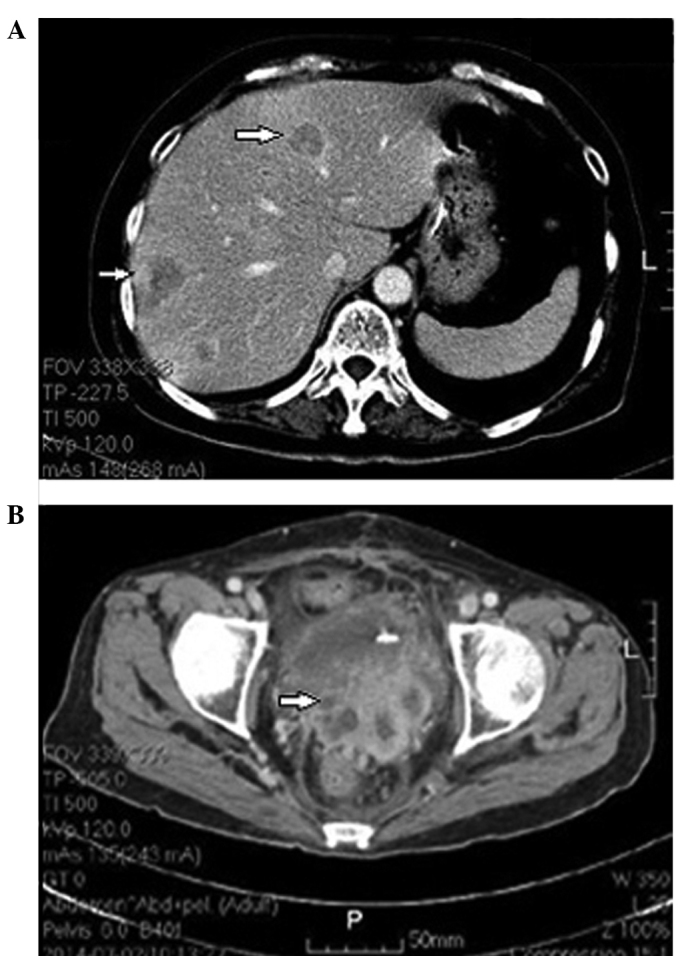
Computed tomography scans revealing multiple metastases in the (A) liver and (B) pelvic recurrence.

Following the surgery, the patient received chemotherapy every 3 weeks for ~6 months. The first chemotherapy cycle was with cisplatin/Taxol (cisplatin, 70 mg/m^2^; Taxol, 150 mg/m^2^), which was followed by 7 cycles of carboplatin/Taxol (carboplatin, AUC = 5; Taxol, 150 mg/m^2^) due to severe vomiting. During chemotherapy, the serum levels of CA19-9 declined to 18.2 U/ml until the eighth cycle. Pelvic CT imaging showed recurrence in the right pelvic cavity, dilatation of the right ureter and right hydronephrosis. Following the ureteroneocystostomy, the patient was planned to receive palliative pelvic external irradiation with a total dose of 45 Gy in 25 fractions, 5 days per week, to eradicate any pelvic residual disease. However, liver metastasis and pelvic progression (Fig. 5), which caused intestinal obstruction, were found after radiotherapy with 10.8 Gy in 6 fractions, and a transverse colostomy was performed. Supportive care, rather than radiotherapy, was provided. After 1 month, the patient succumbed to intestinal bleeding, thrombocytopenia and multiple organ failure.

## Discussion

CUP are defined as a histologically proven metastatic malignant tumors with a poor prognosis, whose primary site cannot be identified following a thorough pre-treatment workup (3). CUP is the seventh most prevalent cancer in the world and the fourth most common cause of cancer-associated mortality in males and females. Among all malignancies, CUP, with a variety of biological characteristics, accounts for 3–5%; ~50% of which are diagnosed as well- to moderately-differentiated metastatic adenocarcinoma, 30% as undifferentiated or poorly-differentiated carcinoma, 15% as SCC and the remaining 5% as undifferentiated neoplasms (4). For SC CUP, the axillary lymph nodes, inguinal lymph nodes, mediastinum and bone are the most common sites of occurrence. The present study reported a rare case of SC CUP with abdominal wall lesions as a primary symptom.

Prior to reaching a diagnosis of CUP, various examinations are required in order to identify the primary site. These examinations should include a detailed medical history, a complete physical examination, including pelvic and rectal examinations, a full blood count and biochemistry analysis, a urinalysis and stool occult blood testing, a histopathological review of any biopsy material using immunohistochemistry, chest radiography, CT imaging of the abdomen and pelvis, and in certain cases, mammography (4,5). In the present case, the patient underwent a laparoscopic left salpingo-oophorectomy 3 months previously, therefore the pathological material obtained was examined section by section using immunohistochemistry to exclude an ovarian origin. In addition, HPV testing, cervical biopsy, gastroscopy and laryngoscopy were performed, all of which were negative. Whole-body fludeoxyglucose-PET/CT imaging has been proven to be useful not only in search for the primary focus, but also for metastases in patients with CUP (1,4,6). Therefore, in order to locate the primary origin, and determine the number of metastases and their locations, the patient in the present study received a PET/CT scan, which demonstrated hypermetabolic lesions in the right pelvic cavity (tumor size, 4.3×2.6 cm) and right inguinal lymph node (tumor size, 0.5×0.9 cm). Histopathological examination of the lymph node revealed moderately-differentiated metastatic SCC.

Routine evaluation of commonly used epithelial serum tumor markers, including CA19-9, CA125, SCC-Ag and CEA, usually have no diagnostic value in identifying the primary site (1,4); however, these markers could be used to predict recurrence and metastasis. In the present case, the pre-operative serum CA19-9 level was elevated at 94.4 U/ml, while the levels of SCC-Ag, CA125, AFP, CEA and NSE were all within the normal reference ranges. During the course of therapy, the serum CA19-9 level was monitored closely; the level decreased to a minimum of 18.2 U/ml and began to rise when the disease progressed and spread to other organs, with a maximum level of 211.1 U/ml. In this case, CA19-9 was an important marker for monitoring tumor recurrence and could be used to evaluate the response to chemotherapy treatment.

CUP remains an extremely aggressive disease with a poor prognosis. The median survival time is between 4 and 12 months (4,7–10), and the 5-year survival rate is <10% (11). Tamam *et al* (6) recorded a median survival time of 9 months for patients with CUP, with a life expectancy between 5 and 25 months, while Fehri *et al* (8) stated that the median survival time was 7 months. In the present study, the tumor progressed rapidly and metastasized to the liver in a short period of time, and the patient succumbed to fever, electrolyte disturbance and malnutrition at 10 months post-diagnosis.

Treatment regimens differ according to the location of the tumor. Although chemotherapy has a role in the treatment of CUP, no regimen has been established as a standard first-line therapy (12). The majority of clinical studies have shown good response rates of 32–55% for cisplatin-based regimens in patients with CUP. According to Nishimori *et al* (13), cisplatin/docetaxel chemotherapy was shown to be effective with tolerable toxicity in patients with CUP. The overall response rate was 62.5% and the median disease-free survival time was 8.7 months. The 1-year overall survival (OS) rate was 68.8%, and the median OS time was 22.7 months. By meta-analysis, Lee *et al* (14) found that platinum-based regimens showed a tendency towards better outcomes compared with non-platinum regimens in terms of survival. The median survival time was 9.4 months, the 1-year survival rate was 36.9% and the 2-year survival rate was 19.7%. Depending on the pathological characteristics, the location of the tumor and the individual performance status (PS), a multimodal therapy that combines cytoreductive surgery, radiation and chemotherapy may be performed in certain cases (15). In the present case, due to the identification of a pelvic mass and affected inguinal lymph node, surgery was performed followed by Taxol and cisplatin/carboplatin treatment. By the 8th cycle of chemotherapy, the serum CA19-9 level had stopped decreasing and began to increase, and pelvic CT imaging showed recurrence in the right pelvic cavity, dilatation of the right ureter and right hydronephrosis. Palliative pelvic external radiation was administered following the ureteroneocystostomy due to residual pelvic lesions; however, the efficacy was limited.

One of the most important targets when treating cancer patients should be prolonging the survival time. Although platinum-based chemotherapy or acceptance into clinical trials may be offered to patients of a relatively young age and good PS, supportive care should be recommended for other patients (16). Following the transverse colostomy procedure in the present case, the majority of treatments were subsequently stopped, with the exception of supportive care. Supportive care in patients with active and incurable SC CUP should be more widely considered.

The prognostic factors reported for CUP, including age, gender, PS, weight loss, pathological subtype, tumor location, number of metastatic sites and serum markers, have been examined in several previous studies (11,17–19). Kodaira *et al* (18) conducted a retrospective analysis for OS in 58 consecutive CUP patients treated with carboplatin plus paclitaxel (Taxol) therapy. The study showed that poor PS, low serum albumin level, pleural effusion, and bone and liver metastases were adverse prognostic factors. In 311 patients with CUP diagnosed in a single university center between 1988 and 2011, analysis of the clinical, pathological and laboratory data led Petrakis *et al* (19) to propose that clinicopathological CUP subgroup and PS were independent prognostic factors. SC CUP with metastasis in the liver has been shown to be associated with a particularly poor prognosis. In a retrospective analysis of 49 patients, the median survival time ranged between 1.7 and 10 months (20). The patient reported in the present study had a good PS, but the pathological subtype was poorly-differentiated SCC with multiple metastases within the pelvic cavity, abdominal wall, inguinal lymph node and liver. The patient survived for only 10 months after the diagnosis.

Additional points also require consideration in the present case study. First, although all the available pathological material from the first surgery was examined and the results were shown to be malignancy-free, the possibility of primary SCC of the ovary could not be completely excluded. In the first surgery, all the resected tissues, including the cyst wall, left ovary and Fallopian tube, were transferred into an internal bag and removed through the 10-mm puncture hole on the right abdominal wall. Due to the large volume, these tissues were cut into several sections while in the bag. Cutting the cyst wall is not optimal for good subsequent pathological examinations, and it is therefore not certain that the complete cyst wall was examined without any omission. Second, the ‘chocolate-like’ fluid that flowed into the pelvic cavity upon breaking of the ovarian cyst may have caused the spread and implantation of potential malignancy, even though the peritoneal cavity was irrigated. During such clinical work, experience from the present case study would maintain the recommendation of using a bag to remove tissues from the abdominal cavity, even in cases where the lesions are considered to be benign. In addition, thorough washing of the abdominal cavity upon tumor rupture, should be performed. During laparoscopic surgery, a method to remove resected tumor tissues that are much larger than the puncture holes should be considered with regard to the requirement for a comprehensive histopathology analysis.

In conclusion, the present study describes a rare case of SC CUP with abdominal wall lesions as the primary symptom. To the best of our knowledge, this is the first case study to have investigated this tumor type. The diagnosis, pathogenesis, treatment and prognosis of this type of tumor has generally been considered poor, thus, additional case reports are required to facilitate the clinical diagnosis and the appropriate course of treatment for affected patients.

## Abbreviations

CUP: carcinoma of unknown primary site
CT: computed tomography
CA19-9: carbohydrate antigen 19-9
CA125: cancer antigen 125
SCC: squamous cell carcinoma
SCC-Ag: SCC antigen
AFP: α-fetoprotein
CEA: carcinoembryonic antigen
NSE: neuron-specific enolase
HPV: human papilloma virus
PET/CT: positron emission tomography/computed tomography
OS: overall survival
PS: performance status
